# Peer Counseling: Method, Metaphor, or Mindset?

**DOI:** 10.3389/fresc.2022.822484

**Published:** 2022-07-22

**Authors:** Leonora Micah Jordan

**Affiliations:** ^1^Department of Social Work, University of Kassel, Kassel, Germany; ^2^Department for Rehabilitation Science, Humboldt University of Berlin, Berlin, Germany; ^3^Center for Playback Theatre, New York, NY, United States

**Keywords:** peer support, independent living, Ergänzende unabhängige Teilhabeberatung – EUTB, Peer Counseling, ableism, counseling methods, expert by experience

## Abstract

Peer Counseling and Peer-to-Peer-Counseling are two common counseling formats worldwide where people meet each other at eye level. Rooted in the Independent Living and Self-Help Movement, “classic” Peer Counseling can be considered a professional counseling service offered by and for people with disabilities. In this article, the question is explored whether Peer Counseling is far more reaching than just being labeled as a counseling method. In many countries, it has now found a permanent place in the counseling landscape among others. Furthermore, the question arises whether it does not also serve as a metaphor for a self-determined way of life. In addition, in this study it will be determined whether and why the mindset and attitude are also important in professional Peer Counseling. Since 2018, Peer Counseling has been offered at over 500 counseling centers in Germany as part of the “Ergänzende unabhängige Teilhabeberatung—EUTB^®^” (Additional Independent Participation Consulting). At a low-threshold and accessible level, advice seekers can find competent individual-centered professional counseling here—often from experts by experience: peers. For many people seeking advice, this is the first and last point of contact for all matters relating to rehabilitation, inclusion and social participation. As part of my PhD-project, I am doing research on Peer Counseling and parallel to this, I train EUTB- and Peer Counselors. Peer Counseling actually is (much more than) a counseling approach which represents a true enrichment for everyone.

## What is Peer Counseling?

“*You want to work? You are blind. You wouldn't even find the office!”*

“*If you want to advise others, then you must take part in specific trainings and exude confidence and competence—how do you want to do that as a wheelchair user?”*

“*You want to counsel others despite your depression? That's not possible—you are mentally ill. You cannot pursue gainful employment in this condition!”*

Similar like this, it may sound when people, who are chronical ill or have a handicap or a participation impairment^1^, tell other people about their career aspirations or future plans.

In March 2009, Germany ratified the UN Convention on the Rights of Persons with Disabilities (UN CRPD); a paradigm shift, manifested in 50 articles from the medical-deficit perspective on dis_abilities^2^ to the establishment of the human rights approach.

Reasons for the development of this convention was the experience of discrimination, disadvantage and exclusion of people with chronic illness and/or dis_abilities worldwide.

Since the 1960's, interest groups and self-advocacy organizations have been campaigning for equal opportunities, the promotion of a self-determined independent lifestyle, non-discrimination, full accessibility and equal participation in social and cultural life. The ratification of the UN CRPD can be identified as a milestone on the way to an inclusive society in which “deviations from the norm” are anticipated as enrichment and diversity.

Peer support services, i.e., forms of mutual support by other persons with disabilities, are cited in Article 26, paragraph 1 of the UN CRPD as a suitable approach to enable persons with dis_abilities to achieve the highest possible degree of self-determination and sovereignty over their own physical, mental-emotional and psychosocial abilities as well as the greatest possible participation in all aspects of life by activating available resources. This can be achieved by the *empowerment approach* used in Peer Counseling [cf. (3), p. 2].

Results of relevant studies point out that Peer Counseling by and for people with dis_abilities can promote the individual's awareness of their abilities, skills, gifts and talents—as required by Article 8 of the UN CRPD [cf. (4, 5)].

The consulting approach of *Peer Counseling* is not new, but has undergone a transformation process since the first utilizations in the 1960's in the USA. Up to a professional counseling offering, which is no longer to be excluded from the counseling landscape of many countries.

**Figure 1:**
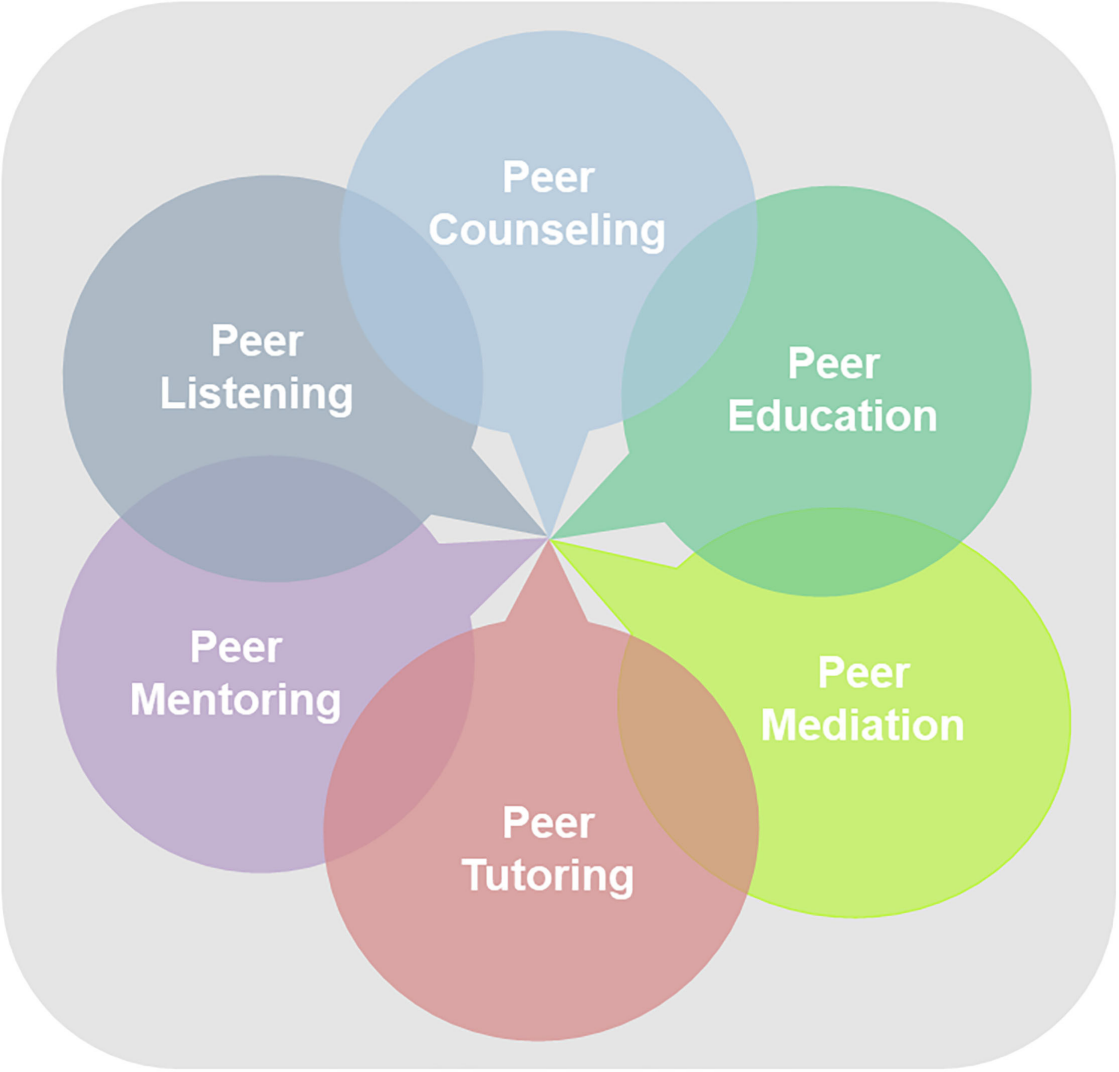
Formats of Peer Support Offerings. In the interest of accessibility, here a complete description of the image: The picture shows a gray square with six circles containing different peer support services. The circles are in semi-transparent colors and overlap partly. With an arrow they all point to the center. On top (on 12 o'clock on a dial) the circle “Peer Counseling”, in the right upper quadrant (2 o'clock) “Peer Education”, in the bottom right quadrant (4 o'clock) “Peer Mediation”, down (on 6 o'clock) “Peer Tutoring”, in the bottom left quadrant (7 o'clock) “Peer Mentoring” and on the upper left quadrant (11 o'clock) “Peer Listening.”

Among other formats of structured and formalized Peer Support Offerings, e.g., Peer Education, Peer Mediation, Peer Tutoring, Peer Mentoring or Peer Listening as pictured on the graph (Figure 1).

Peer-to-Peer Consulting is used in different fields and contexts. *Specially trained peers*^3^ accompany, for example, deployment contingents of the German Army in crisis intervention teams as “*psychological first aiders*” [cf. (6), p. 12–21], can be found in the area of emergency support for civil defense and disaster control forces [cf. (7), p. 42–46], advise students (8), or are involved in education of teachers [cf. (9), p. 74–80].

Whereas, “*classical” Peer Counseling* is a specific counseling service offered *by and for people with dis_abilities*. Here, counseling professionalism and individual biographical experiential knowledge work together [cf. (10), p. 16].

Parallel to other *social movements*^4^, the first Peer Counseling Programs were implemented in the USA in the 1950's. Initially, the focus was on participation programs for seniors [cf. (12), p. 49 f.]. Sponsored by the “Disabled-Student-Program,” in 1962, four students were enabled to study at the University of Illinois and live self-determined—supported by Personal Assistance. In the same year, Ed Roberts sued successfully to the University of California, Berkeley for admission. Shortly afterward other mobility-impaired students, the “Rolling Quads,” followed his example. Those first groups of disabled students initiated to support each other and started empowerment programs for students with participation impairments. Together, they advocated for barrier-free access to university education and self-determined living in the community. As a result, since the 1970's, the “Independent Living Movement” and “Disabled in Action” have been spreading in the USA, advocating for equal participation of people with dis_abilities, offering Peer Counseling, and utilizing the empowerment approach [cf. (13), p. 28–31 and 56–73].

In many countries, anti-discrimination laws have been passed as a result of activities and campaigns by organized self-help and advocacy groups since the 1970's. In Germany, “*Zentren für selbstbestimmtes Leben*” (Centers for Independent Living) have been established since the 1980's. In many places, in addition to barrier-free and accessible contact opportunities and leisure activities, professional qualified advice—Peer Counseling—is also offered by and for people with participation impairments; organized in Germany under the umbrella organization “*Interessenvertretung selbstbestimmtes Leben*—ISL e. V.” [Representation of Self-Determined Life; cf. (14), p. 7].

Since 1994, the “*Bildungs- und Forschungsinstitut für die Selbstbestimmung Behinderter—*bifos e. V.” (Education and Research Institute for the Self-Determination of Disabled Persons) offers annual training courses for Peer Counselors. The curriculum is based on the curriculum developed by the US Independent Living Movement. Other self-advocacy associations have designed curricula to qualify people with various forms of participation impairments as Peer Consultants as well:

- “*EXperienced-INvolvement—Genesungsbegleitung”* (EXperienced-INvolvement Recovery Support): Since 2005, people with a psychiatric diagnosis have been trained nationwide as EX-IN-Recovery-Supporters. More than 500 EX-IN-Supporters advise people with psychiatric illnesses on various topics—such as structuring everyday life, resilience, resource orientation, and dealing with medication.
  Some are offerering consultation hours in psychiatric clinics, others are affiliated with self-help associations and initiatives^5^.
- “*Blickpunkt Auge-Beratung”* (Eyepoint Eye-Consulting): The “*Deutsche Blinden und Sehbehinderten Verband—DBSV e. V*.” (German Association for the Blind and Visually Impaired) has also been conducting training courses at various locations by and for blind and visually impaired people since 2012. Providing comprehensive and independent advice to other affected people, their relatives and interested parties on topics relating to vision (loss), eye diseases and visual impairments as well as the provision of aids and appliances^6^.
- “*Beratung auf Augenhöhe”* (Eyelevel Consulting): In various model projects, people with so-called intellectual disabilities have been enabled by “*Lebenshilfe Service gGmbH”* at different locations since 2013 to advise other people with learning difficulties on the topics of housing, work, and leisure time^7^.
- *DeafMentoring*: As the name suggests, in first line this Peer–Consulting-Offer is located in the area of mentoring programs. Deaf and severely hearing impaired people accompany other deaf people as mentors on their way into professional life, aligned at Rheinisch-Westfälische Technische Hochschule Aachen^8^.

With the nationwide introduction of the “*Ergänzende unabhängige Teilhabeberatung—EUTB*^®^” (Additional Independent Participation Consulting) in Germany in 2018, the offering structures of Peer Counseling shall also to be strengthened [cf. (15), p. 12 f.].

In many EUTB-offices, people with chronic illness and/or dis_ability are employed; some as full-time counselors, several on a part-time basis, and about one third of the EUTBs employ Peer Counselors on voluntary basis. Since, according to the mandate, advice is given on all questions of rehabilitation, inclusion and social-cultural participation, it is not least the people seeking advice who benefit from the heterogeneous composition of the advice teams (4).

**Figure 2:**
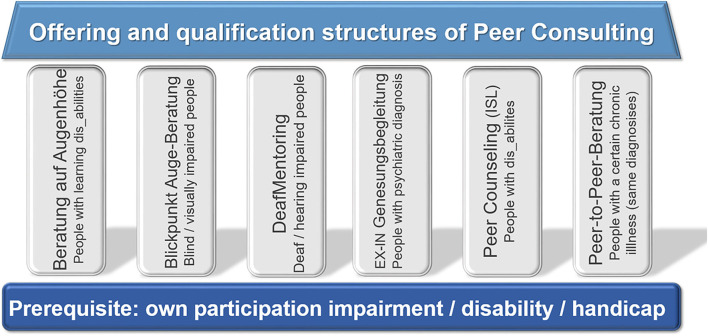
Offering and qualification structures of Peer Consulting in Germany. Pictured is a graphic that resembles a house in its structure. At the bottom of the picture—corresponding to the foundation of a building –is a transverse bar, titled “Prerequisite: own participation impairment / disability / handicap.” Based on this, there are six pillars that carry the roof “Offering and qualification structures of Peer Consulting.” Each of these six pillars represents an approach to Peer Consulting Training: From left to right there are: “*Beratung auf Augenhöhe*”—Eyelevel Counseling by and for people with learning disabilities, “*Blickpunkt Auge-Beratung*”—Eyepoint Eye-Consulting by and for blind and/or visually impaired people, “*DeafMentoring*”—a mentoring program, by and for deaf and/or hearing impaired people, “*EX-IN-Genesungsbegleitung*”—EX-IN-Recovery-Support by and for people with psychiatric diagnosis, *Peer Counseling* by and for people with disabilities, “*Peer-to-Peer-Beratung*”—Peer-to-Peer-Counseling by and for people with a certain chronic illness (same diagnosis).

In Germany, the diverse range of professional peer-consulting-offers rests on several pillars, as pictured in the model (Figure 2).

Even if the preparatory qualifications for Peer Counselors and Peer Consultants are neither standardized nor uniform, it is still possible to speak of *professional counseling*. Following the *extended concept of profession*, Peer Counselors are trained *to act professionally*, learn to *apply conversation and communication techniques* appropriate to the situation and addressee, have a broad (experiential) knowledge, organize the counseling sessions in a *formalized setting*, and there is a clear dividing line between counselor and counselee: Here in the form of a knowledge advantage between those seeking advice and those providing it.

## Peer Counseling: Only a Counseling Method?!

In the relevant literature, Peer Counseling is referred to as “*the* pedagogical method” of the Independent-Living-Movement. As accessible voluntary counseling offered by and for people with participation impairments in all four dimensions^9^—content, spatial, social, and temporal—the “*Berufsverband für Peer Counseling—BVP e. V.”* [Professional Association for Peer Counseling; cf. (17)] is oriented toward approaches and methods that are also used in other counseling and consulting offers.

Traditionally, Peer Counseling follows an *integrative approach* to counseling based on a humanistic view. According to the person-oriented empowerment concept, Peer Counseling takes place in a context- and issue-specific on all topics that the counselees bring to the table [cf. (17, 18), p. 43 f., (19), p. 20–31]:

- active listening and questioning,
- client-centered conversation according to Carl Rogers,
- resource and social space orientation
- systemic interventions,
- solution-focused approaches, such as Personal Future Planning, and
- following the basic attitude of empathy, acceptance, and authenticity.

In principle, Peer Counseling is free of charge counseling service, and independent of economic, business, or budgetary interests [cf. (20)]. Although, Peer Counseling is—even if the counselors are “only” engaged in voluntary or part-time work—a (partially) formalized *professional counseling service*. Information transfer and accompanying support, based on conversations and other (crisis-) intervention techniques, take place in a protected setting in an appealing atmosphere. The roles are clearly defined in advance: There is the qualified Peer Counselor and the person seeking advice (counselee); both encounter each other on an equal footing, unlike is sometimes the case with professional consulting services offered by government agencies and authorities, coaching or psychotherapy.

Peer Counseling—following the idea of a low-threshold and accessible open setting—is mostly oriented toward a “come structure,” i.e., the counselees decide the topics and contents to be discussed (*participatory justice*). In addition to leading the conversation and clarifying concerns, Peer Counselors tend to take the role of *experienced process facilitators*. Due to their reflected professional biographical (dis_ability) experiences, they can help to shape the counseling relationship and process with their expertise, but they do not bear the responsibility for successful crisis management, solution development, or implementation of new knowledge [cf. (21, 22), p. 52 ff., (23), p. 74–85].

Rather, it is the concern and mission of Peer Counselors to (re-)discover individual resources together with the counselees, to support them in decision-making and problem-solving processes—always with the aim that the counselees (re-)gain self-determination and the ability to act for living in independency.

Current research shows that, in the context of Peer Counseling, a significant role can be attributed to the shared lifeworld, similar life realities, and the shaping of relationships. Aspects of this social, societal, and at the same cultural construction of reality are depicted in the approaches of *interactionist constructivism* according to Reich [(24), p. 121 f.]. In particular, interaction at the peer *and* counseling relationship level makes it possible to initiate reflection and learning processes in a targeted manner and to facilitate changes in perspective at a low-threshold appreciating level.

Personal development limits can be recognized and overcome in direct peer-to-peer contact during counseling. Here lies a great opportunity in the *uniqueness of the peer moment* in the professional setting of Peer Counseling.

In addition to *consulting-specific knowledge* of counseling methods, approaches, and techniques, Peer Counselors have specific professional and *content-related field knowledge* and are located “in the group” of people with chronic illness and/or dis_ability who, despite all the challenges, have achieved certain basic stability and self-determination in their personal way of life. They may not necessarily be older in terms of years of life than the counselees, but they do have a head start in terms of knowledge and competence, more experience in dealing with authorities, and can draw on a supportive network of colleagues.

A differentiated professional and informal knowledge as well as experience and action knowledge form, in addition to a reflective collegial exchange—ideally supplemented by supervision, intervention, or case and team discussions—a secure basis for professional Peer Counseling, in order to develop adequate approaches to solutions together with the counselees [cf. (21), p. 274 f., (25)].

For many concerns, Peer Counseling Services can be the first and central point of contact, in addition to professional competence, psycho-emotional support with a respectful welcoming culture in the sense of the empowerment. An appreciating approach and deep inside-understanding encounter at eye level alone can encourage counselees to become active themselves, to stand up for themselves and their own needs, and to want to take control of their own lives (again). Peer Counselors can be irreplaceable guides and companions in this process.

## Peer Counseling—Attitude and Mindset?!

People with a chronic illness and/or dis_ability still experience paternalism, pitying looks, derogatory remarks, and exclusion in everyday life; they are often not expected to lead a self-determined independent life, their ability to make decisions is denied and their failure to conform to physical and psychological norms is viewed with a deficit-oriented eye—as recited in the introducing sentences at the beginning. Statements like these are familiar to many Peer Counselors as well as to people with dis_abilities and/or chronic illnesses.

It is precisely the *reductionist medical view* of (socially undesirable) deviations from norms that makes it difficult to develop a positive self-image, value one's own body as “beautiful” or realize one's own resources, gifts, and talents.

Of course, there are impairments and dis_abilities which do affect the achievement of performance standards. Of course, not all impairments can be “treated away” and full recovery is not always possible. It cannot be wiped aside that impairments hinder everyday life or in certain situations. But it is important to overcome obstacles and find a good way to deal with existing and emerging barriers.

It is precisely at this point that Peer Counseling can intervene with its empowerment approach: Consistently, the focus here is on the individual and not on the impairments, deficits, or deviations. Rooted in the self-help and self-advocacy movement, Peer Counseling counters experiences of devaluation and demoralization with proven strategies, measures, and concepts in addition to active listening.

On an *intra- and interpersonal level*, Peer Counseling can help people to (re)find a reconciliatory way of dealing with their own body and lovingly accept it despite its deviations from the norm, to (re)discover inherent resources, talents, and gifts, to find a new way of dealing with an illness and to accept the limitations that come with it. As well as realizing that the counselees are not alone in their situation and that life with the existing impairment can still be enriching, worth living, and enjoyable.

As mentioned above, the UN CRPD is based on a human rights understanding of dis_ability and the idea of an inclusive diversity-oriented society. Explicit reference is made to the active participation of people with dis_abilities in political decision-making processes. Societal and socio-political changes arise through the activism of social movements, this is also due to the commitment of self-advocacy and self-help groups. People who come together to form networks of peers—occasionally encouraged by Peer Counselors can give their interests considerably more emphasis, communicate them to third parties and actively represent them externally.

In fact, people—encouraged by empowerment processes in Peer Counseling—become “experts for their own life situations.” Self-aware, they can succeed in standing up for their personal needs with an open view and healthy self-confidence, or in asserting interests together as a group in solidarity with peers [cf. (26)].

The outcome of Peer Counseling was demonstrated in a study of people with mental illness in the early 2000's in the USA: The rehabilitation sociologist Salzer (27) describes that those people who go through a peer program learn to deal with their illness in a positive way.

The Peer Counselors are not only perceived as hopeful role models, but this “upward comparison” leads to an increase in self-confidence in one's own abilities, which can lead to empowerment processes (*social comparison theory*). Similar results have also been obtained by researchers who have evaluated different Peer Counseling Programs [cf. (4, 5, 26, 28)].

Peer Counselors can be attributed—partly explicitly, partly implicitly—to have the *function as role model* in different ways. Not least because counselees meet peers in a professional setting who are engaged in a regular (gainful) activity or occupation despite/because of living with a chronic illness and/or dis_ability. Plus, the participation impairment itself is anticipated as (only) a part of identity.

Central methods of professional Peer Counseling approaches are actually exemplified and related to one's own person/personality development. Therefore, empowerment processes have a circular effect in the context of Peer Counseling.

**Figure 3:**
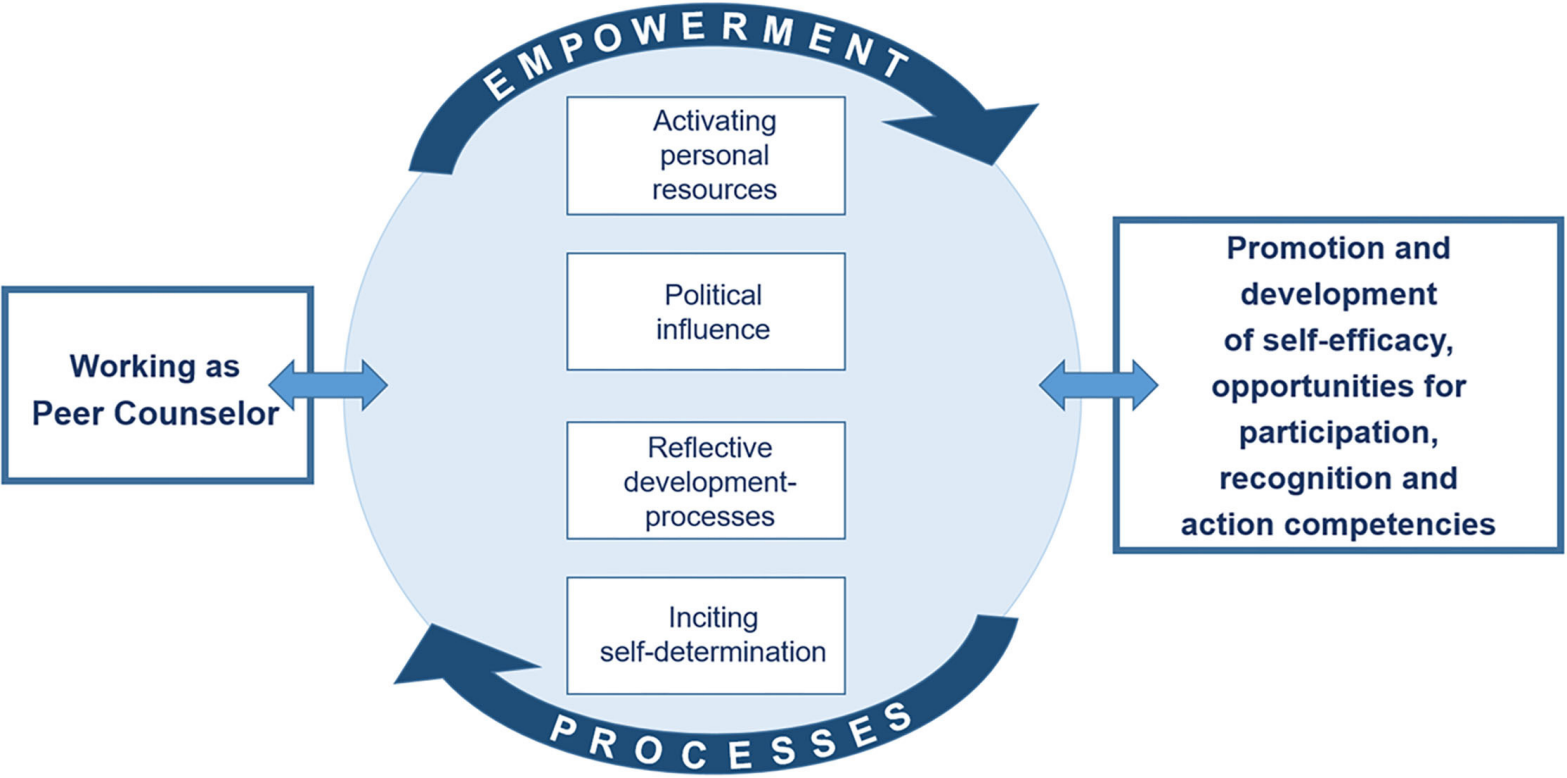
Circle of empowerment in Peer Counseling. Shown here is a circle which contains four terms in rectangles. From top to bottom: “Activating personal resources,” “Political influence,” “Reflective development-processes,” and “Inciting self-determination.” Above this circle, is an arrow in which “Empowerment“ is written—pointing clockwise, and below, the arrow picks up the direction with the word “Process.” To the left (on 9 o'clock), next to the circle, is a rectangle that reads “Working as a Peer Counselor.” On the opposite side, at 3 o'clock, it reads “Promotion and development of self-efficacy, opportunities for participation, recognition and action competencies.”

Working as a Peer Counselor might include activating personal resources of counselees and counselors, has a political influence, enables reflective development processes and is inciting self-determination. Empowerment-effects of Peer Counseling can be: promoting and developing self-efficacy, providing opportunities for participation, recognition and enhancing action competencies; illustrated in the following model (Figure 3).

## Conclusion

Peer Counseling, as a professional counseling service offered by and for people with chronic illness and/or dis_ability, can contribute at the socio-political level to ensure that people with participation impairments also receive attention and consideration in the political arena. Representatives of umbrella organizations are, for example, heard as experts in legislative procedures, are active in advisory committees, and within the framework of the nationwide offer of “*Ergänzende unabhängige Teilhabe-beratung—EUTB*” (Additional Independent Participation Consulting) both, the Peer Counseling format and Peer-to-Peer-Consulting approaches find an equal place in the professional counseling landscape in Germany.

The qualifying training for Peer Counselors are conducted by experienced Peer Counselors who often also have a relevant academic degree and are employed in counseling professions, e.g., as a psychotherapist, social worker, educational consultant, teacher, coach, lawyer, or similar.

Many Peer Counselors are also involved in self-advocacy and self-interest associations and advocate for an inclusive society with equal education and opportunities through their work, but also actively demand equal participation in all aspects of social and cultural life [cf. (26)].

On an *individual level*, Peer Counseling can enable people to cope with crisis situations in life in a self-determined way, to mobilize their own abilities and resources to overcome challenges, find a reconciliatory way of dealing with their own illness/dis_ability and be valued as a person.

Although Hermes [(25), p. 8 f.], Wienstroer [(29), p. 179 ff.], and van Kan [(20), p. 17 ff.] refer to *Peer Counseling as an independent method* in contrast to psychotherapy and other counseling and consulting formats, different principles of action are intertwined in “classic” Peer Counseling:

- **Personal life-world (dis_ability) experiences:** The common backexperience of living with a chronic illness and/or dis_ability—regardless of the type and extent of the form of participation impairment—forms a trust-building basis for the encounter in Peer Counseling. Qualified Peer Counselors can encourage counselees to come to terms with their dis_ability-related effects, inform them about practical support options for a self-determined lifestyle, to point out choices and various options for taking responsibility in dealing with crises.
  Through their commitment (and their way of life), Peer Counselors might also be seen as role models: They succeed in motivating counselees extrinsically and intrinsically to lead a life on their own, formulate goals, and work toward achieving them.
-**Partiality:** In Peer Counseling, the counselors use their competencies and expertise in the interest of the counselees. Since both actors are people with participation impairments, counseling concerns can be perceived, analyzed, and comprehended from a professional meta-level as well as from an internal perspective.
  In contrast to other counseling formats, Peer Counselors can identify with many issues and concerns in different qualities, because they themselves may have already dealt with similar situations or have been confronted with comparable challenges.
-**Holism:** Counselees can inquire about Peer Counseling with all concerns about all questions of lifestyle, dealing with their dis_ability, rehabilitation-possibilities, and participation issues. Different approaches and methods are used in counseling processes in order to understand and accompany the counselees in the best possible way.
  In accordance with the bio-psychosocial model of the International Classification of Disabilities and Health (ICF), the individual life situation, personal views, and interests, environmental and personal resources, goals, and needs of the counselee are always included in the decision-making and solution-finding processes in professional Peer Counseling.
- **Emancipation:** Influenced by the historically conditioned medical-deficient perspective on dis_ability and the associated equation of illness and dis_ability with the need of care and welfare, it was and is a concern of Peer Counseling—in close connection with interest and self-advocacy associations—to appeal to the autonomy, judgment, decision-making, and agency of people with participation impairments.
  Based on the assumptions of the empowerment principles, every person has abilities, talents, gifts, and resources. Peer Counseling can support other people in knowing these and using them in a meaningful way to realize the own dreams and future perspectives, in order to stand up for own interests and needs with healthy self-confidence.

These described resource-oriented approaches of Peer Counseling can support individuals as well as them as a “group of people with chronic illness and/or dis_ability” to overcome patronizing, excluding, and stigmatizing structures.

This specific counseling service represents also an opportunity to offer marginalized people, at a low-threshold level, an accessible space where their concerns are heard. People in crisis situations, for example in the midst of sudden and profound changes in entire life situation due to an illness or (newly acquired) dis_ability, find competent and understanding contact persons in a timely manner. Aware of their function as guides and their competencies, Peer Counselors can also refer to other institutions or services, if needed or wanted.

Despite possible proximity due to a similar illness or dis_ability, qualified Peer Counselors must find a balance in the counseling relationship. In addition, professional field competencies complement their own reflected biographical experiences, so that only specific and in individual cases do Peer Counselors incorporate personal coping strategies into counseling processes. For the most part, “classic” elements of communication-, interviewing- and intervention techniques are used to exploit the possibilities of the empowerment approach.

Peer Counseling, with the factual and technical competencies taught in training courses, counseling-specific methodology, and the underlying approaches of empowerment, is much more than just a target group-specific professional counseling format. Performance-oriented use of methodology, professional biographical competencies, and the personal prerequisites, as well as the fact that Peer Counselors are themselves people with a participation impairment, distinguish this unique counseling format.

In a mutually complementary way, Peer Counseling can be understood not only as an indispensable additional counseling method but also as a tool for self-help and self-advocacy. The theory-based approaches described above are also reflected in the values and action convictions by Peer Counselors themselves.

With the implementation of more than 500 counseling centers of “*Ergänzende unabhängige Teilhabeberatung—EUTB*” (Additional Independent Participation Consulting) throughout Germany, there is an opportunity to create nationwide structures of professional Peer Counseling and Peer-to-Peer-Consulting Services. The draft “one for all” can be implemented in a truly low-threshold and accessible manner in the sense of lived participation in a diversity-oriented society.

Who can advise more professionally and comprehensively on questions in the field of participation opportunities, rehabilitation, or social inclusion than qualified peers? Who—in addition to relevant qualifications—is having suitable experiences themselves? Not always academic degrees from the profession (orientation), but also biographical knowledge and preparatory courses can lead to special skills and expertise.

At this point, answers to the questions formulated at the beginning of this article can be found: In Peer Counseling, counselees can be given competent and comprehensive advice on planning and organizing everyday life issues, individual career perspectives, the application of remedies and aids, educational and occupational opportunities and dealing with illness and dis_ability; on the other hand, there is the possibility—assuming interest and suitability—of taking training courses to become a Peer Counselor oneself, in order to support other people in similar situations in the future. Peer Counseling can be clearly understood as a highly specialized counseling method, as a metaphor for emancipation and self-determination, and as an underlying attitude and mindset.

## Author Contributions

The author confirms being the sole contributor of this work and has approved it for publication.

## Conflict of Interest

The author declares that the research was conducted in the absence of any commercial or financial relationships that could be construed as a potential conflict of interest.

## Publisher's Note

All claims expressed in this article are solely those of the authors and do not necessarily represent those of their affiliated organizations, or those of the publisher, the editors and the reviewers. Any product that may be evaluated in this article, or claim that may be made by its manufacturer, is not guaranteed or endorsed by the publisher.

## Footnotes

^1^In this article, the terms *participation impairment, handicap, impairment*, and *dis_ability* are used synonymously. This refers to people with one (or more) chronic illnesse(s), existing and/or officially recognized (severe) dis_ability(ies)—regardless of the type and extent of the disability(ies) because for participation in Peer-Counseling-Training-Courses, the *form of participation impairment* is subordninated.

^2^The spelling of dis_ability with an underscore (*ability gap*) points out that the transitions to “non-disability” are partly fluid, change in different phases of life and are not an obstacle/barrier per se in everyday life and situation. Rather, disability represents a partly medical, partly structural, and partly socially constructed category that favors the exclusion of people with physical or psychological “deviations from the norm” through attributions or the design of architecture [cf. (1), p. 1]. In addition, the ability gap points out that there is no such thing as “*the* disabled person/s,” since people who have the same diagnosis, for example, deal with symptoms in different ways. This is depending on the manifestations, individual life situation, the individual living environment, as well as other personal conditions. In *Intersectionality Research* as well as in the emancipatory-participatory oriented *Disability Studies*, disability is understood as a socio-cultural process of categorization and stigmatization. Therefore, the ability gap visually forms a reference to these relations of difference and inequality of a hierarchical-normative social order [cf. (2), p. 38].

^3^*Peer* means “of equal rank, equivalent, similar interests.” In this context, the peer-concept aims at the fact that both the counselor and the counselee are, for example, in a similar life environment (professional affiliation, biographical experience or else) or both are in a comparable (life) situation or – as found in “classical” Peer Counseling”—both are people living with a chronic illness and/or dis_ability.

^4^Like “need-based” associations, *social movements* have sociopolitical, economic or cultural origins. The intention is to work toward changes in the public and political sphere in order to assert interests, intentions and goals in politics and society. Examples of those civic emancipation movements are: anti-nuclear movement, labor movement, civil rights movement, peace movement, women's rights movement, student movement, veterans' movement, or the disability movement [cf. (11), p. 13–60].

^5^Further information online: https://ex-in.info; last reviewed on: 19.10.2021.

^6^Further information online: https://blickpunkt-auge.de; last reviewed on: 19.10.2021.

^7^Further information: https://www.lhbl.de/peercounseling-116.html?file=files/lebenshilfe/pdf-anlagen/art ikel-360-peercounseling.pdf and online at: https://www.inform-lebenshilfe.de/inform/veranstaltungen/ter mine/bv/210253-bv-peer-unterstuetzung.php; last reviewed on: 19.10.2021.

^8^Further information online: https://signges.rwth-aachen.de/go/id/ljbg; last reviewed on: 19.10.2021.

^9^These four dimensions of low-threshold accessible (Peer-) Counseling Offers are described in the literature as follows: In terms of *content and concept* (open setting, openness to all topics and concerns), in the area of *spatial accessibility* (accessible without steps or obstacles, easy to find with good local transportation connections, and appropriate equipment), *social low-threshold* (no administrative hurdles, protecting the anonymity of counselees, counseling is free of charge), and the *temporal* dimension [clearly communicated office and consultation hours as well as time-independent options for making contact and prompt allocation of appointments; cf. (16), p. 98–106].
